# Capacity building of nurses providing neonatal care in Rio de Janeiro, Brazil: methods for the POINTS of care project to enhance nursing education and reduce adverse neonatal outcomes

**DOI:** 10.1186/1472-6955-11-3

**Published:** 2012-03-12

**Authors:** Brian A Darlow, Andrea A Zin, Gina Beecroft, Maria EL Moreira, Clare E Gilbert

**Affiliations:** 1 Department of Paediatrics, University of Otago, Christchurch, New Zealand; 2 Paediatric Ophthalmologist, Instituto Fernandes Figuera, FIOCRUZ, Rio de Janeiro, Brazil; 3 Neonatal Service, Christchurch Women's Hospital, Canterbury District Health Board, Christchurch, New Zealand; 4 Neonatologist, Instituto Fernandes Figuera, FIOCRUZ, Rio de Janeiro, Brazil; 5 International Centre for Eye Health, London School of Hygiene and Tropical Medicine, London, UK

**Keywords:** Brazil, Neonatal care, Neonatal nursing, Quality improvement, Neonatal mortality, Premature infant, Retinopathy of prematurity, Education, Continual professional development

## Abstract

**Background:**

Increased survival of preterm infants in developing countries has often been accompanied by increased morbidity. A previous study found rates of severe retinopathy of prematurity varied widely between different neonatal units in Rio de Janeiro. Nurses have a key role in the care of high-risk infants but often do not have access to ongoing education programmes. We set out to design a quality improvement project that would provide nurses with the training and tools to decrease neonatal mortality and morbidity. The purpose of this report is to describe the methods and make the teaching package (POINTS of care--six modules addressing **P**ain control; optimal **O**xygenation; **I**nfection control; **N**utrition interventions; **T**emperature control; **S**upportive care) available to others.

**Methods/Design:**

Six neonatal units, caring for 40% of preterm infants in Rio de Janeiro were invited to participate. In Phase 1 of the study multidisciplinary workshops were held in each neonatal unit to identify the neonatal morbidities of interest and to plan for data collection. In Phase 2 the teaching package was developed and tested. Phase 3 consisted of 12 months data collection utilizing a simple tick-sheet for recording. In Phase 4 (the Intervention) all nurses were asked to complete all six modules of the POINTS of care package, which was supplemented by practical demonstrations. Phase 5 consisted of a further 12 months data collection. In Phase 1 it was agreed to include inborn infants with birthweight ≤ 1500 g or gestational age of ≤ 34 weeks. The primary outcome was death before discharge and secondary outcomes included retinopathy of prematurity and bronchopulmonary dysplasia. Assuming 400-450 infants in both pre- and post-intervention periods the study had 80% power at *p *= < 0.05 to detect an increase in survival from 68% to 80%; a reduction in need for supplementary oxygen at 36 weeks post menstrual age from 11% to 5.5% and a reduction in retinopathy of prematurity requiring treatment from 7% to 2.5%.

**Discussion:**

The results of the POINTS of Care intervention will be presented in a separate publication.

**Trial registration:**

Current Controlled Trials: ISRCTN83110114

## Background

As neonatal care develops in countries with emerging economies it has been common to observe increasing survival but also increased morbidity among survivors. Retinopathy of prematurity (ROP), one of the major morbidities following preterm birth, has become a significant cause of blindness in children in middle income countries in Latin America, Asia and Eastern Europe. Gilbert and colleagues have called this the "third epidemic" of blindness due to ROP [1,2] being a mixture of first epidemic risk factors (uncontrolled use of 100% oxygen) and second epidemic risk factors (increased survival of extremely low birth weight babies). In these countries, unlike in the established market economies, some babies affected by severe ROP have relatively high gestational age and birthweights (that is > 30 weeks and/or > 1250 g respectively) and it is clear that many cases are preventable with more optimal neonatal care.

Despite improvements in many health indicators, the proportion of preterm deliveries in Brazil has increased from just 4% in the early 1980s to more than 10% after 2000 [3]. Although many of these preterm births are 34 week's gestation or more and birthweight over 2000 g, the number of infants at risk of ROP have considerably increased over this period [3].

Nurses have a key role to play in the care of high-risk and preterm infants. However, many countries have a severe shortage of qualified nurses and a great deal of care is administered by nurse assistants or auxiliary nurses (NAs), who may have only minimal training. There is often a lack of ongoing education programmes for nurses and NAs and, in addition, many neonatal intensive care units (NICUs) lack protocols for common care practices. Zin has previously reported that the incidence of severe ROP (ROP needing treatment according to the Early Treatment for Retinopathy of Prematurity trial recommendations) in 7 NICUs in Rio de Janeiro varied from 2.1% to 7.8% and that NICUs with the lowest rates had more optimal nurse to patient ratios [4].

In a national study involving all New Zealand infants with birthweight < 1500 g admitted to a neonatal unit in 1986, the two largest hospitals had both the lowest mortality and lowest morbidity, including rates of ROP, after adjustment for birthweight and gestation [5]. Although there are a number of possible explanations for this finding, one possibility is that these hospitals were able to deliver overall better care. The authors proposed that ROP might be a good index of overall quality of care.

We therefore hypothesised that providing nurses/NAs with a focused education package and strengthening the capacity of nurse supervisors, as well as supplying minimum essential equipment, in neonatal units in Rio de Janeiro, Brazil, would improve survival and decrease morbidity, particularly ROP. Further, we considered that the training package would improve current nurse/NA practices and empower them to undertake additional responsibilities for babies in their care where these were supported by their medical colleagues.

The aim of this report is to describe the methods used in this quality improvement project, and to make available the interactive training package called POINTS of Care.

**Ethical approval **was obtained from the London School of Hygiene & Tropical Medicine and, in Rio de Janeiro, the Ethical Review Board from Secretaria Municipal de Saúde, the Regional Health Authority, and the Brazilian Ministry of Health's Ethics Committee (CONEP). Informed consent was obtained from all health personnel involved in the study. Informed consent was not required from mothers of preterm babies, as only routine audit data were collected and used in the analyses.

## Methods/Design

Six government funded NICUs, caring for 40% of preterm infants with BWs of ≤ 1500 g born in Rio de Janeiro in 2008, were invited to take part. Five of these NICUs had been included in Zin's previous prospective study of ROP [4,6]. They all had existing ROP screening and treatment programs and they were willing to participate.

**Figure 1:**
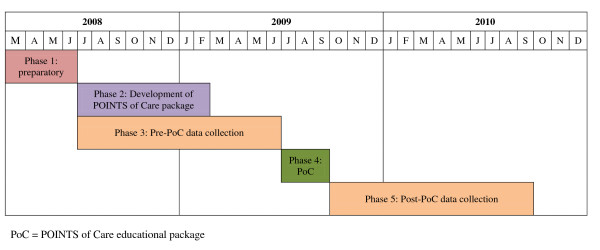
**Time course of the POINTS of Care project**.

Our original plan was to adopt an interrupted time series (ITS) methodology, that is to introduce the intervention in NICUs A and B for three months with NICUs C-F as controls, then add the intervention in NICUs C and D for three months and finally E and F for three months. However, during our initial NICU visits we learned that many nurses/NAs worked in more than one NICU in our study, which would have led to contamination. We therefore altered the design to a controlled before-and-after (CBA) study with data collection for 12 months, an educational intervention package over three months, followed by a further 12 months data collection (Figure 1).

### Phase 1: Preparatory phase (4 months)

During this initial phase of the study multidisciplinary workshops were held in each of the six NICUs to identify outcomes of interest, to decide upon methods of data collection and to undertake a situation analysis with respect to staffing levels and availability of protocols for care and essential equipment. Head nurses, registered nurses, NAs, and some ancillary health professionals and neonatologists/residents attended these workshops.

During the workshops the major findings from Zin's previous study of ROP were presented by way of introduction. Following this process it was agreed to focus assessment on outcomes among very low birthweight infants only (VLBW: birthweight ≤ 1500 g and/or gestation ≤ 34 weeks). In addition to mortality and ROP, unit staff identified outcomes as being important to them. Infection, necrotizing enterocolitis and neonatal chronic lung disease were morbidities that were of particular concern. Staff also wished to evaluate breast feeding, nutritional support and achievement of adequate growth.

As there was no systematic way of collecting daily information prior to the start of this study, a daily "tick sheet" was introduced, based on that used in Christchurch, New Zealand, after modification by neonatologists and nurses in Rio. NICUs agreed that data would be collected by one to two senior staff in each NICU daily during the study (Additional file 1).

A standard reporting form was used to collect information on staffing levels, and on the availability of functioning equipment, for example air-oxygen blenders, vital sign monitors and pulse oximeters. Structured sheets were designed to record observation of nurse's practices before and after training, and this was piloted to test suitability.

A data collection form for the outcomes of interest was developed and pilot tested. Data were extracted by a trained Research Assistant from the tick sheet summaries, discharge summaries, medical records and ophthalmologists' records.

### Phase 2: Development of intervention

During the interactive workshops in each NICU in Phase 1, care teams were asked to identify major concerns and education gaps so that an intervention strategy could be tailored to the overall needs of the NICUs. At the end of this process it was agreed that the intervention package would be as follows: self-guided use of educational materials, a DVD with practical demonstrations and provision of minimum essential equipment. Design, development and pilot testing of the educational component of the intervention took place at the same time as the 12 month pre-intervention period of data collection (Phase 3--see below). The topics addressed comprised **P**ain control; optimal **O**xygenation; **I**nfection control; **N**utrition interventions; **T**emperature control; and **S**upportive care, which we called "**POINTS **of Care" (see Additional files 2, 3, 4, 5, 6, 7 and 8). These topics were all approved by local senior neonatologists and management.

Six mini-courses were developed, one for each of the POINTS of Care educational package. As far as possible the main elements of each education package were strongly evidence based and reflected material based on Cochrane reviews (when available) [7] and the in-house protocol handbook of the Neonatal Intensive Care Unit, Christchurch Women's Hospital, Christchurch, which is reviewed annually. Each mini-course comprised pre-course questions, guiding principles, facts about the topic, and then repeated questions so that nurses could assess what they had learned. After going through the educational materials, nurses were asked to make suggestions to improve care in their NICU.

Each NICU had a verbal presentation of one of the POINTS of Care mini-course to assess the content, comprehension and local relevance before the final versions were produced. Practical demonstrations were also undertaken jointly by a nurse educator from New Zealand (GB) and two experienced local nurses to refine the DVD content.

Alongside the written material a DVD was developed that gave a practical demonstration of one aspect of each of the POINTS of Care mini-courses. This DVD came with an Instructors booklet giving a clear learning objective for each video clip. The nurse educator from New Zealand used this material to train the local nurse educators. The clinical scenarios covered were as follows: Pain--a baby having a heel prick blood test demonstrating the beneficial effects of oral sucrose; Oxygen--illustrating the importance of setting and responding to saturation alarms; Infection--illustrating how work surfaces can become contaminated; Nutrition--skin to skin contact whilst having a nasogastric tube feed; Temperature--a preterm baby having a bath; Supportive care--a baby receiving nasal CPAP having routine care and positioning. A small, pocket sized booklet with a summary of the main principles and facts of each mini-course on one double-sided, laminated page was also designed so that each nurse and NA could have a personal copy at all times. All educational material was developed in English and translated into Portuguese.

A questionnaire was developed to assess nurse's knowledge before and after the training package and this was pilot tested.

### Phase 3: Pre-intervention period (12 months)

Data were collected using the tick sheets, discharge summaries and other data sources for the period July 2008 to June 2009 inclusive. In addition, all NICUs were visited monthly during this period by one of two senior local nurses who made unannounced visits at different times of the day. They supervised data collection and were available to assist with any difficulties with the process. During these visits standardized observations were made on medical and staffing levels; whether the unit was overcrowded; the availability of essential consumables and the proportion of babies on supplemental oxygen who were being monitored. They observed and recorded hygiene behavior of nurses and checked the proportion of monitors which had alarms that were correctly set. They also observed and recorded how nurses responded to alarms going off for up to five babies receiving supplemental oxygen, and recorded the temperature on arrival in the NICU of the last five babies admitted.

### Phase 4: Delivery of the intervention (3 months)

After discussion it was agreed that formal teaching of all qualified nurses and NAs on all six NICUs in all six POINTS of Care was not practical or feasible. All nurses and NAs were, therefore, asked to work through each mini-course in their own time, or working in groups, to earn a certificate. Written course material was supplemented by the DVD, with these practical demonstrations being undertaken by one of the two local nurses who helped develop the content. (See Summary on this page.) In all cases this process was supported by the Head Nurse of the NICU. Neonatologists were requested to read the mini-courses so that they were familiar with the material and could answer any questions from the nurses.

Further interactive workshops were held in each NICU after completion of the POINTS of Care module so that nurses/NAs could identify potentially better practices to introduce to their unit. Key items of equipment (e.g. saturation monitors and probes, blenders etc.) identified as priority needs, were itemized at this time and ordered (within budget constraints) by the end of 2008.

### POINTS of Care Summary

1. POINTS of Care training packages (see Additional files). Every nurse and NA was asked to work through each of the six mini-courses, answer a series of question pre-course, read the educational material, answer the same questions post-course and make suggestions on any practice changes that might be appropriate for their NICU. On completion of all six mini-courses participants were issued with a certificate of achievement.

2. Interactive workshops in each NICU to identify potentially better practices that could be integrated into everyday care of that unit and identification of essential items of equipment

3. Questionnaires to all nurse/NAs to assess their attitudes to the course

4. Nurse educator "champions" visited each NICU monthly to reinforce concepts taught in the mini-courses and hold practical sessions using the DVD.

### Phase 5: Post intervention data collection period (12 months)

Data collection continued after the intervention exactly as in Phase 3 for a further 12 months from October 2009 to September 2010 inclusive. Visits by one of the two experienced local nurses continued at about monthly intervals during this period to reinforce POINTS of Care training and to audit NICU practices.

### Outcomes

The overall aim of this project was to reduce mortality and morbidity, specifically bronchopulmonary dysplasia (BPD), severe ROP, sepsis and necrotising enterocolitis (NEC), and to improve the nutritional status of premature babies being cared for in NICUs in the government sector in Rio. Outcomes were limited to babies who were inborn on the study units and who had birthweights of ≤ 1500 gs or gestational age of ≤ 34 weeks.

The primary outcome was death before discharge.

Secondary outcomes were:

• Death before discharge, by BW categories

• Retinopathy of prematurity: a) Type 1 ROP or treatment of ROP (Type I ROP defined as: Zone I, ROP stage 1-2 with Plus; Zone I, ROP stage 3 with or without Plus; Zone II, ROP stage 2-3 with Plus) [8] and b) ROP of any stage

• Bronchopulmonary dysplasia a) oxygen required at 36 weeks post-menstrual age (PMA), for babies < 32 week's gestation b) oxygen required at 28 days of age and c) oxygen required at 28 days of age, by birthweight

• Necrotising enterocolitis (clinical diagnosis with radiological evidence of pneumatosis or pneumoperitoneum)

• Sepsis: a) early-onset sepsis: blood culture positive sepsis within 48 h of birth; b) late-onset sepsis (suspected): clinical sepsis after 48 h of age and treated with antibiotics for seven days and c) late-onset sepsis (culture positive): as above but with a positive blood culture. Both suspected and culture proven late-onset sepsis will be reported as a) percentage of admissions with one or more episodes, and b) number of sepsis episodes per 1,000 baby days.

• Days to regain BW

• Change in nurses' knowledge of key elements of neonatal care, as measured by questionnaire before and after undergoing the training package

• Change in nursing practices, assessed by observation during unannounced visits

• Nurses' satisfaction with educational package, assessed by a structured questionnaire

### Power calculations

From exisiting data, it was anticipated that approximately 400-450 babies with BWs < 1,500 g would be admitted to the study units each year. A study with a one year pre-intervention period and a one year post intervention period would be adequately powered to detect the following differences, at 80% power and at *p *= < 0.05: an increase in survival from 68% to 80%; a reduction in BPD (oxygen at 36 weeks post menstrual age) from 11% [9] to 5.5% and a reduction in ROP requiring treatment from 7% to 2.5%.

### Data management

Data on the clinical outcomes were entered into a database created in Access by an experienced, trained research assistant. The quality of data entry was checked by the Principal Investigator, who cross-checked data from randomly selected data forms against that entered into the database. Standard procedures were used to clean the data; for example, frequency distributions and cross tabulations with review of the data collection form for outliers. Data were transferred into STATA for analysis. Data from the other data sources (such as questionnaires administered to nurses; observation of nurses practices) were entered into further databases created in Access, or into Excel spreadsheets. These data sources were analysed in Excel (nurse observation) or in STATA (comparison of nurses knowledge and practices before and after undergoing POINTS of Care training).

## Results

The results of the POINTS of Care intervention will be presented in a separate publication.

## Discussion

There is a great deal of evidence from high quality randomized clinical trials and systematic reviews on the efficacy of a wide range of interventions in relation to the care of preterm babies [7,10]. However, as in all areas of public health, there is often a gap between this evidence base and the delivery of interventions in the real world [11,12]. This has led to considerable discussion and debate concerning how complex interventions, which may entail improving the knowledge, skills and attitudes of staff, and interventions to improve the functioning of other aspects of the health system (for example, health management information systems; leadership and governance) can best be evaluated [11,13,14].

As with new drugs or medications, randomized clinical trials provide the highest level of evidence and this is also true of the evaluation of complex interventions. However, evaluation of complex interventions is complicated by the need to attribute the inputs to the outputs, outcomes and ultimate impact of the intervention on health. Evaluation of changes to the health system, such as training staff and providing additional equipment, also needs to take account of, and measure if possible, unintended positive or negative consequences, as well as the impact of extraneous factors, such as a policy change, or change in the environment external to the heath system, such as extremes of climate. In relation to nursing interventions for the prevention of morbidity in preterm infants being cared for in intensive neonatal care units, the evidence of impact is limited [15-18].

When the results of this study are published we will discuss the findings in relation to the challenges and constraints of delivering and evaluating complex interventions, highlighting the difficulties of attributing inputs to outcomes and measures of impact.

## Abbreviations

ROP: Retinopathy of prematurity; NICU: Neonatal intensive care unit; NA: Nurse assistant or auxillary nurse; VLBW: Very low birthweight; CPAP: Continuous positive airways pressure; BPD: Bronchopulmonary dysplasia; NEC: Necrotizing enterocolitis; BW: Birthweight; PMA: Post-menstrual age.

## Competing interests

The authors declare that they have no competing interests.

## Authors' contributions

BD contributed to the project design, developed the system for data collection and the Points of care teaching module and wrote the first draft of the manuscript. AZ jointly conceived the project as a response to findings of her PhD, contributed to the project design, helped refine the teaching modules and critically reviewed the manuscript. GB helped develop the Points of care teaching module and trialled its effectiveness, and critically reviewed the manuscript. MM contributed to the project design, obtained ethics approval in Brazil and support of the study hospitals, helped refine the teaching modules and critically reviewed the manuscript. CG jointly conceived the project having supervised AZ's PhD, was PI on applications for funding, obtained ethical approval in London, helped refine the teaching modules and critically reviewed the manuscript. All authors approved the final (submitted) manuscript.

## Pre-publication history

The pre-publication history for this paper can be accessed here:

http://www.biomedcentral.com/1472-6955/11/3/prepub

## Supplementary Material

Additional file 1 **PoC Tick sheet**. Click here for file

Additional file 2 **PoC Instruction for nurses**. Click here for file

Additional file 3 **PoC Mini-course on pain in the newborn**. Click here for file

Additional file 4 **PoC Mini-course on management of oxygen in babies with respiratory distress**. Click here for file

Additional file 5 **PoC Mini-course on newborn infection**. Click here for file

Additional file 6 **PoC Mini-course on newborn nutrition**. Click here for file

Additional file 7 **PoC Mini-course on temperature control in the newborn**. Click here for file

Additional file 8 **Mini-course on supportive care in the newborn**. Click here for file
