# Supplementary material for: Capacity building of nurses providing neonatal care in Rio de Janeiro, Brazil: methods for the POINTS of care project to enhance nursing education and reduce adverse neonatal outcomes
Source: BMC Nurs. 2012 Mar 12;11:3. doi: 10.1186/1472-6955-11-3 (PMC3395837; doi:10.1186/1472-6955-11-3)
Supplement: Additional file 1 — PoC Tick sheet. [file 1472-6955-11-3-S1.PDF]

1. Unit: ______________________ **2. Study Number** ☐☐☐☐

3. Mothers name / sticker ______________________ 4. Hospital number: ____________

5. Date of birth ☐☐ *Day* ☐☐ *Month* 2 0 ☐☐ *Year* 6. Sex: ☐ M ☐ F

7. Date admitted ☐☐ *Day* ☐☐ *Month* 2 0 ☐☐ *Year* 8. Birth weight ☐☐☐☐ gs

9. Date discharged home/died/ transferred ☐☐ *Day* ☐☐ *Month* 2 0 ☐☐ *Year* 10. Gestational age: ☐☐ . ☐ weeks

11. ≤1,500g and/or ≤34 weeks ☐ Yes ☐ No

		**Jan 21**	**Jan 22**	**Jan 23**	**Jan 24**	**Jan 25**	**Jan 26**	**Jan 27**	**Jan 28**	**Jan 29**	**Jan 30**	**Jan 31**	**Total**
12. Baby transferred	Now												
13. Oxygen	Now												
14. Intubated	Now												
15. CPAP	Now												
16. Hood/nasal catheter	Now												
14. Saturation monitor in use	Now												
15. Parenteral nutrition	Now												
16. Nasogastric tube	Now												
17. Umbilical artery catheter	Now												
18. Umbilical venous catheter	Now												
19. Peripheral IV	Now												
20. Long line (PICC)	Now												
21. Mothers milk	Last 24hr												
22. Donor milk	Last 24hr												
23. Formula feed	Last 24hr												
24. Blood culture taken	Last 24hr												
25. Positive blood culture	Last 24hr												
26. Ampicillin given	Last 24hr												
27. Gentamicin given	Last 24hr												
28. Other antibiotic given	Last 24hr												
29. Blood transfusion	Last 24hr												
30. Caffeine/theophylline	Last 24hr												
31. Glucose/sucrose used	Last 24hr												
32. Kangaroo care	Last 24hr												

Comments: ______________________________________

____
