# Supplementary material for: Capacity building of nurses providing neonatal care in Rio de Janeiro, Brazil: methods for the POINTS of care project to enhance nursing education and reduce adverse neonatal outcomes
Source: BMC Nurs. 2012 Mar 12;11:3. doi: 10.1186/1472-6955-11-3 (PMC3395837; doi:10.1186/1472-6955-11-3)
Supplement: Additional file 2 — PoC Instruction for nurses. [file 1472-6955-11-3-S2.DOC]

# POINTS of Care Training Course for Nurses

The **POINTS** of Care training course for nurses is made up a package of 6 self study mini-courses. The topics of the mini courses are:

**P p**ain prevention and management

**O o**xygen management and respiratory distress

**I i**nfection control

**N n**utrition optimisation

**T t**emperature control

**S s**upportive care

These are key areas of care that all nurses working in neonatal units should develop knowledge and skills in and which form the foundation on which we build neonatal care and from which we can further develop nursing practice.

Each mini-course has a small set of questions at the beginning, to encourage you to think about what you already know on the topic. This is followed by some information about the topic. The same questions are then repeated again at the end of the information section.

The idea is to read the questions first, answer them as honestly as possible then read the information. This is to allow you to give your thoughts about the topic. Once you have read the information, answer the same set of questions again at the end to see if it confirms your thoughts or has given you some new ideas or knowledge.

In addition there is section at the end where you can write down your own suggestions for you own work setting if the topic has given you any ideas that you think might be useful to help improve the care your neonatal unit delivers to neonates and their families.

When you complete one mini-course your answered questions and suggestions will be collected in. Marks will not be given for the “right” answers but we ask that you try and answer all the questions to achieve a certificate showing you have completed the mini-course.

It is anticipated each mini-course will take about 30-40 minutes to complete. A set of suggested answers to the questions and references will be kept with your course supervisor if you do wish to check your answers when you have completed all the mini courses.

Also, in addition to a certificate for completing each mini course, when you have completed all six courses you with receive a set of POINTS of Care mini cue cards that highlight some of key points about each topic.

If you have any questions please do not hesitate to contact your course supervisor. Name:

Thank you very much for your time and effort.
