# Supplementary material for: Capacity building of nurses providing neonatal care in Rio de Janeiro, Brazil: methods for the POINTS of care project to enhance nursing education and reduce adverse neonatal outcomes
Source: BMC Nurs. 2012 Mar 12;11:3. doi: 10.1186/1472-6955-11-3 (PMC3395837; doi:10.1186/1472-6955-11-3)
Supplement: Additional file 3 — PoC Mini-course on pain in the newborn. [file 1472-6955-11-3-S3.PDF]

MINI- COURSE

on

PAIN in the NEWBORN

<u>Instructions:</u>

Read each sheet and answer any questions as honestly as possible

The first sheets have five questions to allow you to give your feelings about pain in newborn babies

The next sheets you some information about pain in the newborn

- *What causes pain*
- *Why pain is important and how to detect pain*
- *How pain might be prevented*
- *How pain might be treated*

The five questions are then repeated. We will not be giving marks for "right" answers but do ask you to answer all the questions to achieve a certificate showing you have completed this ***Mini-Course***

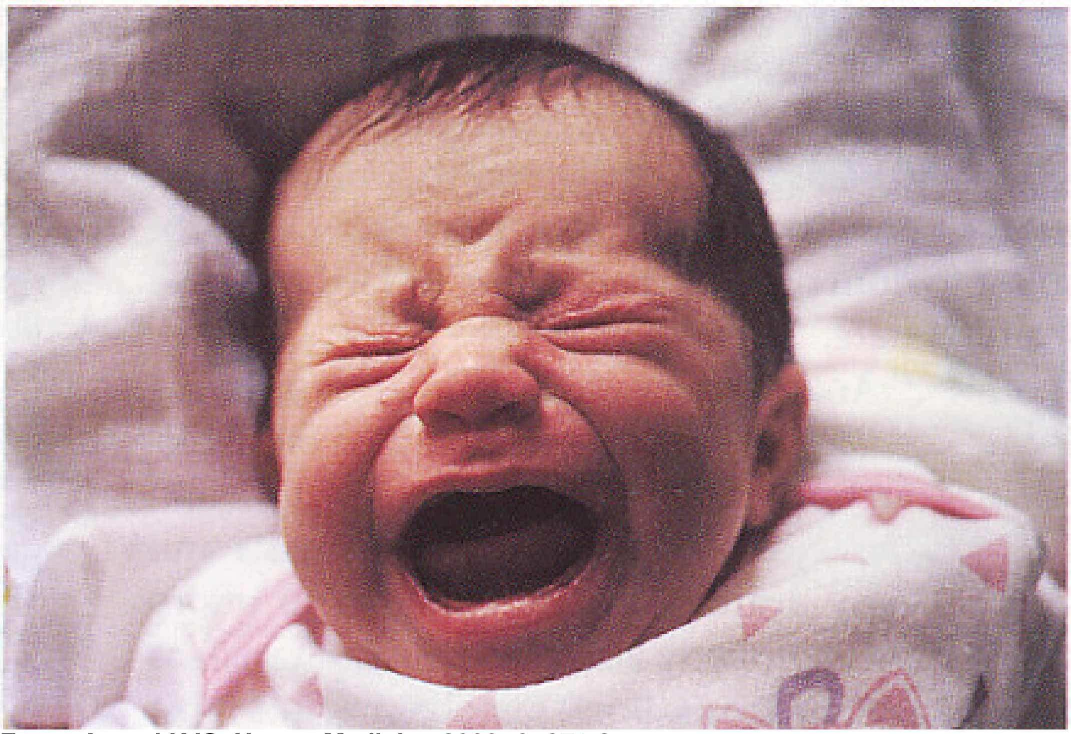

From: Anand KJS. Nature Medicine 2000; 6: 971-3

Is this infant in pain?

If you think “yes” – why?

What other signs of pain might there be?

What things might be painful for babies?

How can you tell if a baby is in pain?

How can pain be avoided?

Common false beliefs

- “Preterm infants don’t feel pain”

- “Preterm infants don’t remember pain and it has no long term effects”

- “It is too dangerous to give anaesthesia or analgesia”

Guiding Principle

Neonates, term and preterm, do experience pain and have the right to receive effective and safe pain relief

What things might be painful for babies?

Some common painful procedures include:

Heel prick
Arterial or venous line insertion
Inserting naso-gastric tube
Intubation
Chest drain insertion
Pulling off tape!

Can you think of others?

How can you tell if a baby is in pain?

We all react to pain with behavioural, physiological and biochemical and hormonal changes.

A behavioural response might be a withdrawal reflex.

A physiological response might be an increase in blood pressure.

Infant pain scales combine a number of measures to give an objective assessment of pain.

How can pain be avoided?

Avoid unnecessary procedures

Anticipate episodes that might be painful

Act to prevent or minimise pain

- *Non-pharmacological*
- *Pharmacological*

PAIN SCALES

Use of pain scales:

- heightens awareness of pain in babies
- encourages anticipation of painful episodes – and ways of avoiding or minimising these
- allows babies to have appropriate analgesia

SUN SCALE (Modified) for Assessing Neonatal Pain

Compared with two other scores (NIPS and Comfort), the Scale for Use in Newborns (SUN) * *was preferable because of its ease of use, scale symmetry and scoring consistency.*

*Adapted from Blauer T. Clin J Pain 1998; 14: 39-47

The SUN Scale has 5 parameters, each scored 0 - 2

There are 3 behavioural categories:

1. Facial expression

2. CNS state

3. Movement

And 2 physiological parameters:

4. Breathing

5. Heart rate

The baby's baseline heart rate should be recorded

1. Facial expression

Normal, relaxed	*0*
Increased tension, furrowed brow	*1*
Furrowed brow, tightly closed eyes, grimace, vigorous cry	*2*

2. Central Nervous System State

Asleep or awake, quiet, calm	*0*
Anxious, fussy	*1*
Hyper-alert, panicked	*2*

3. Movement

Relaxed, normal tone	0
Intermittent increased activity, flexion and extension of extremities	1
Frequent flexion and extension or flaccid, minimal movements	2

4. Breathing

Quiet resps, relaxed normal pattern.<br>*If intubated, synchronised*	0
Intermittent increased rate >60.<br>*If intubated frequent non-synchrony*	1
Frequent increased rate >60.<br>*If intubated, fighting ventilator*	2

5. Heart rate

Baseline (record)	0
Elevation 10% - 15%	1
Elevation >15%	2

Record Total Score

Usually treat with score of 4 or more

Record intervention

Containment, touch

Non-nutritive sucking

Oral sucrose

Other - record

PAIN RELIEF or PREVENTION

1. Non-pharmacological

   - Reduce noise / light

   - Positioning / swaddling
     Kangaroo care is very good – even for babies on nCPAP (good for mothers and fathers too)

   - Sucking / pacifier

   (Attach pulse oximeter before anticipated painful episodes)

2. Sucrose (Glucose or expressed breast milk)

- RCTs support effectiveness prior to a range of painful procedures, including in preterm infants

- *Wide range of strengths used; 24% to 67%*

- *Wide range of doses; 0.05ml to 2ml*

- In term babies we use 0.5ml aliquots

- <30 weeks, we use 0.05ml aliquots

3. Other

Paracetamol (acetominophen)

Rectal
20 mg/kg/dose, 6 -12hrly up to max 80 mg/kg

Oral
10 -15 mg/kg/dose, 4 –12hrly up to max 60 mg/kg

Consider morphine

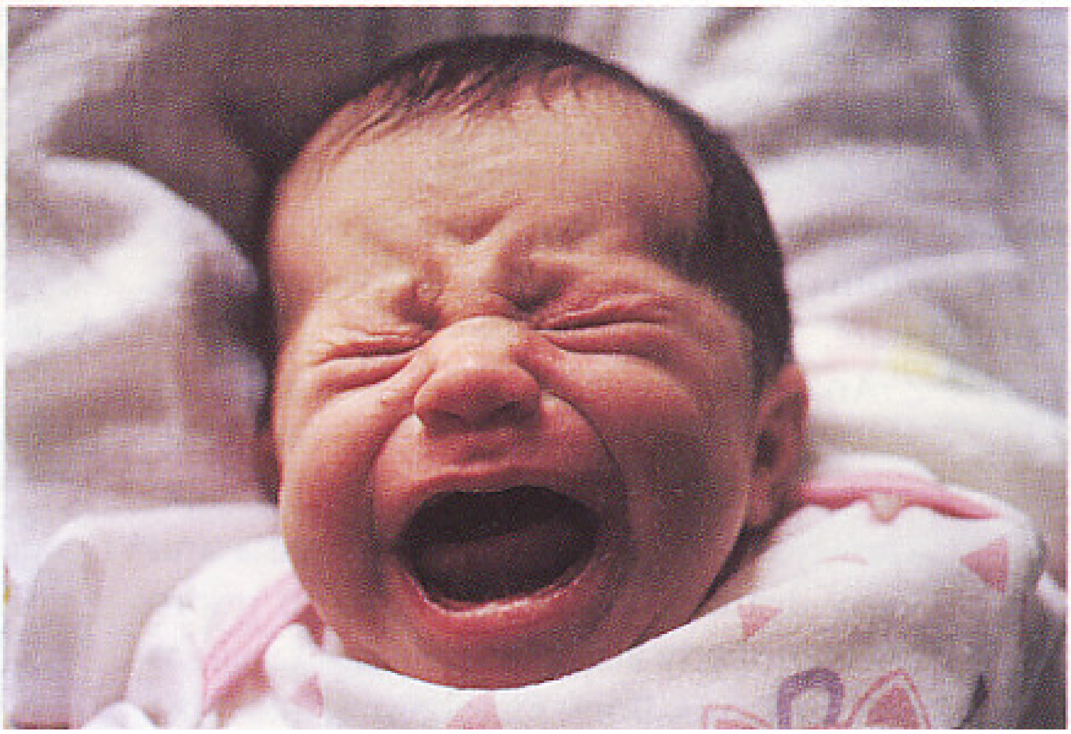

Is this infant in pain?

If you think “yes” – why?

What other signs of pain might there be?

What things might be painful for babies?

How can you tell if a baby is in pain?

How can pain be avoided?

Are there 3 or 4 practical things you could suggest which may help how newborn pain is managed in your nursery?
(Please list these)

(These suggestions will go into a book for all the staff to consider)

THE END – THANK YOU
