# Supplementary material for: Capacity building of nurses providing neonatal care in Rio de Janeiro, Brazil: methods for the POINTS of care project to enhance nursing education and reduce adverse neonatal outcomes
Source: BMC Nurs. 2012 Mar 12;11:3. doi: 10.1186/1472-6955-11-3 (PMC3395837; doi:10.1186/1472-6955-11-3)
Supplement: Additional file 4 — PoC Mini-course on management of oxygen in babies with respiratory distress. [file 1472-6955-11-3-S4.PDF]

MINI- COURSE
on
Management of OXYGEN in babies with RESPIRATORY DISTRESS

Instructions:

Read each sheet and answer any questions as honestly as possible

The first sheets have 5 questions to allow you to give your thoughts about newborn respiratory distress

The next sheets give you some information about newborn respiratory distress

- *Oxygen delivery systems*
- *Why oxygen may be harmful*
- *How best to monitor oxygen delivery*
- *Treatment of apnoea of prematurity*

The five questions are then repeated. We will not be giving marks for "right" answers but do ask you to answer all the questions to achieve a certificate showing you have completed this ***Mini-Course***

What different ways do you know to deliver oxygen to a baby with respiratory distress?

What harm can be caused by not giving enough oxygen?

What harm can be caused by giving too much oxygen?

How can the amount of oxygen the baby is getting be monitored?

What is “apnea of prematurity” and how can we treat it?

Common false belief

"Oxygen is good for you, so more must be better"

Guiding Principle

Oxygen is the commonest "drug" we give to preterm infants – we should be as careful with the amount we give as with any other drug.

What ways are there to give oxygen to babies with respiratory distress?

The most effective way of giving oxygen to a baby will vary with the cause and severity of their respiratory distress.

The simplest way of giving supplementary oxygen is via a **head-box** or **oxy-hood.** Most commonly 100% oxygen is fed into a perspex box or hood around the baby's head. If there are few gaps around the baby the oxygen concentration may reach 60% or more. It is useful to use an **oxygen analyser** in the hood to see how much oxygen the baby is being given. With this way of delivering oxygen the baby does all the work of breathing.

Nasal continuous positive airways pressure (nCPAP) is usually given via a single nasal prong or paired nasal prongs. The system delivers both oxygen and pressure and the latter can reduce the work of breathing. CPAP can be particularly useful in hyaline membrane disease where the lungs' air sacs (alveolae) tend to collapse.

In industrialised countries, the gas used to deliver CPAP is a mixture of air (21% oxygen) and 100% oxygen. Mixing occurs in a "**blender**" which is set at the oxygen % wanted.

Babies who have great difficulty breathing may be helped by a **ventilator**, which delivers oxygen to the baby via an **endotracheal tube**. Nearly all ventilators will have a built-in air-oxygen blender so that anything from 21% to 100% oxygen can be given.

A useful way to give oxygen is via a **nasal cannula**. A thin tube delivers oxygen to the nose, either by small holes in the tube below the nostrils or by small prongs in each nostril.

Although a nasal cannula can be used with an air-oxygen blender, most frequently 100% oxygen is used. However, the amount of oxygen delivered to the baby will depend on the flow rate and the weight of the baby:

Estimated oxygen concentration:

> *If flow (litres per min) exceeds weight (in kg) the concentration will be 100%*
>
> e.g. A 1 kg baby receiving 1 litre/min will be getting 100% oxygen.
>
> A 2 kg baby will need a flow rate of 2 litres/min to get 100% oxygen.
>
> Tables are available to estimate oxygen concentrations delivered at lower flow rates.

What harm is caused by too little oxygen?

We depend on oxygen to keep us alive and maintain brain function and the function of other organs.

The fetus develops in a low oxygen environment – oxygen has to get from the mother’s lungs via her blood stream and cross the placenta to reach the fetus. The oxygen saturation in the mother will be close to 100% but in the fetus it is more like 70%. But the fetus does not need much oxygen to fuel the work of breathing and exercise, and to digest food, because the mother is doing this on its behalf.

After birth the preterm baby will need more oxygen than it has been getting *in utero*. If the oxygen is too low it is possible the baby may not survive or that he/she will have some brain damage.

What harm is caused by too much oxygen?

Oxygen is like all good things, it is possible to have too much of it. Think of sunlight; we need it but too much will cause sunburn.

As oxygen is used by the body, tiny amounts of compounds called "free-radicals" are produced. These compounds have an unpaired electron and can damage surrounding molecules and structures. We have several protectors in the body against these damaging compounds. These protectors are enzymes and also some of the vitamins we get every day, particularly vitamin A, C and E.

For a healthy adult breathing air (21% oxygen) the protectors can easily deal with the harmful compounds. But if we were to breathe 100% oxygen then the protectors would be overwhelmed in about 24 hours and we would start to feel unwell.

Because the fetus is in a low oxygen environment, it has low levels of these protectors, but their levels rise a few weeks before the baby is due to be born at full term (40 weeks). If the baby is born prematurely it still has low levels of protectors but it also often needs extra oxygen because the lungs are immature. So preterm babies are particularly at risk from too much oxygen.

Oxygen free radicals contribute to:

Lung damage – **Chronic lung disease**
Eye damage – **Retinopathy of prematurity**
Gut damage – **Necrotising enterocolitis**
Brain damage – **Periventricular haemorrhage**

and may affect **growth and development**

Oxygen is the commonest “drug” we give
to preterm babies

We should be as careful with the
amount of oxygen
we give as with any other drug

MORE is NOT BETTER

How can we monitor how much oxygen the baby is getting?

This is the oxygen saturation curve. It relates the partial pressure of oxygen (pO_2), which determines how much oxygen gets to the tissues, to the saturation (SaO_2)

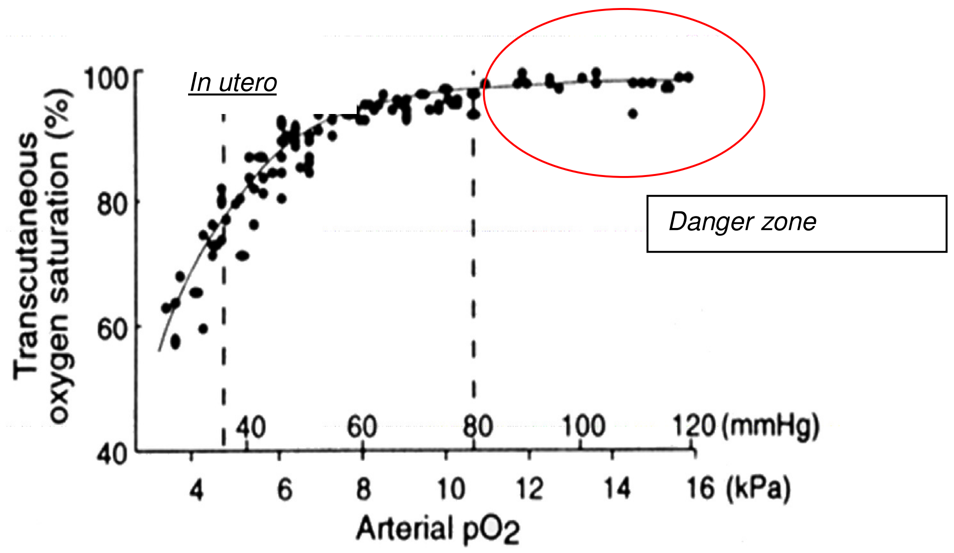

Adapted from: Richmond S. Clin Perinatol 2006; 33: 11-27.

In utero, the saturation is about 50-70% and the pO_2 is 30-35 mmHg.

After birth, if the saturation is over 95%, the arterial pO_2 may be very high, increasing the risk of damage from oxygen.

Actually we do not know for certain what levels of oxygen are too low or too high for preterm babies – several studies are underway around the world to try and find this out.

In the meantime most experts aim for a saturation (SaO_2) in preterm babies between 85% and 95% and suggest 88%-92% might be optimal.

From the diagram you can see there are two ways of measuring oxygen in the baby.

We can measure oxygen tension (pO_2) by performing a "blood gas" on an arterial oxygen sample. Getting this sample may be painful for the baby so the sample may not be that accurate. Sometimes we want to know the pH and the pCO_2 as well and this can be done from a heel prick.

Continuous oxygen monitoring can be:
by **transcutaneous oxygen,** which estimates the pO_2 (here the target may be 50-75 mmHg).

or by a **pulse oximeter** / **saturation monitor,** which gives the SaO_2 . Because this is non-invasive it has become the commonest way to monitor.

As noted above the preferred target at present
is **88%-92%.**

Oxygen saturation monitoring

- In preterm babies in oxygen, oximeters should be used when possible
- It is more important to use an oximeter when the baby is unwell or being handled
- It is more important to use an oximeter when a baby is in >30% oxygen

PRETERM BABIES ALMOST NEVER NEED
100% OXYGEN

Suggestions for how to react to Oxygen alarms

1. When saturation target is **88% - 92%** the suggested alarm limits are:
   Lower alarm limit at 86%
   Upper alarm limit at 94%

2. If the baby is in air (21% oxygen), and the saturation reading is 96%-100%, it is permissible to switch the upper alarm off.

   Remember to switch the alarm on again if the baby is in added oxygen again.

3. When in added oxygen, minimise % time at 96%-100%.
   Aim for 88%-92%.

4. Do not "Titrate" FiO2 (extreme fluctuations in SpO2 are risky). Allow baby to vary within the desired saturation parameters.

5. In sustained hyperoxia, make small adjustments of 2-5% in the FiO2 and remain at the bedside until the situation is stabilised.

6. In sustained desaturation, assess the baby for central cyanosis, bradycardia, or poor perfusion before making any change. Desaturation without bradycardia is less significant than desaturations with bradycardia. Always stay with the infant until saturations stabilise in an acceptable range.

   Make small increases in FiO2 of 2-5%, reassessing the baby after each step. If there is poor improvement despite a large increase in FiO2, call a doctor for help.

Apnea of prematurity

Apnea in the newborn is defined as cessation of breathing for 20 seconds or more, accompanied by a colour change and bradycardia – a heart rate of less than 100.

Apnea of prematurity is more common with decreasing gestation, 3/4 of all babies of less than 28 weeks gestation will have apnea, and 1/2 of those of 31-32 weeks gestation. It is uncommon beyond 35 weeks gestation.

There are many causes of apnea in the newborn and babies who start having apnea for the first time usually need some investigations – particularly to rule out infection and anaemia.

Treatment of primary apnea
The simplest way to stop an apneic episode is to stimulate the baby.

Treatment with methylxanthines – theophylline or caffeine – is very effective but these drugs tend to increase the heart rate and cause gastro-oesophageal reflux.

Nasal CPAP is also a useful treatment.

There is some evidence that giving supplementary oxygen makes apnea less frequent and the episodes less severe. However, there are many dangers in treating apnea just with extra oxygen because when the baby is well – not apneic – it is very likely to be in too much oxygen. Oxygen saturation monitoring should always be used if possible.

What different ways do you know to deliver oxygen to a baby with respiratory distress?

What harm can be caused by not giving enough oxygen?

What harm can be caused by giving too much oxygen?

How can the amount of oxygen the baby is getting be monitored?

What is “apnea of prematurity” and how can we treat it?

Are there 3 or 4 practical things you could suggest which may help how oxygen therapy is managed in your nursery?
(Please list these)

(These suggestions will go into a book for all the staff to consider)

THE END – THANK YOU
