# Supplementary material for: Capacity building of nurses providing neonatal care in Rio de Janeiro, Brazil: methods for the POINTS of care project to enhance nursing education and reduce adverse neonatal outcomes
Source: BMC Nurs. 2012 Mar 12;11:3. doi: 10.1186/1472-6955-11-3 (PMC3395837; doi:10.1186/1472-6955-11-3)
Supplement: Additional file 5 — PoC Mini-course on newborn infection. [file 1472-6955-11-3-S5.PDF]

MINI- COURSE

on

NEWBORN INFECTION

Instructions:

Read each sheet and answer any questions as honestly as possible

The first sheets have four questions to allow you to give your thoughts about infection in babies

The next sheets give you some information about newborn infection

- *How common is newborn infection*
- *The outcomes of newborn infection*
- *The sources of newborn infection*
- *How best to prevent newborn infection*

The four questions are then repeated. We will not be giving marks for "right" answers but do ask you to answer all the questions to achieve a certificate showing you have completed this ***Mini-Course***

Which is more common
– early-onset sepsis (that is infection in the first 48 hours of life),
or late-onset sepsis (that is infection after the first 48 hours)?

Where do the organisms causing late-onset sepsis come from?

What is the outcome from late-onset sepsis?

How can late-onset sepsis be prevented?

Common false belief

"If your hands look clean, they will not pass on infection"

Guiding Principles

Preterm babies are much more susceptible to infection than adults and are less able to combat such infection.

HAND WASHING, before and after handling every baby, is the most important way to prevent cross-infection.

How common is sepsis?

By sepsis or infection, we generally mean **"bacterial infection in the blood stream"** but there can be infection in the CSF, the renal tract, the lungs (pneumonia), as well as inflammation of the bowel (necrotising enterocolitis - NEC) and at other sites. There can also be local infection, such as a skin abcess.

The incidence of neonatal sepsis with a positive blood culture is often reported related to 1,000 live births. In industrialized countries it is estimated that between 1 and 8 infants per 1000 births will have systemic sepsis in the first month of life. Preterm infants and low birthweight infants have a higher incidence of sepsis than infants born at term.

The incidence of sepsis in neonatal units in developing countries can be very high – as much as 30-60% of all admissions.

Early-onset sepsis is usually defined as proven infection within 48 hours of birth, with the source of infection generally being from the maternal vaginal tract.

The organisms most commonly associated with early onset sepsis are *Group B Streptococcus* (GBS) and Gram-negative organisms, particularly *Escherichia coli* (E. coli).

These organisms colonize the vagina and cause infection in the baby by first invading the lining of the uterus, then in turn infecting the amniotic fluid, the lungs of the fetus and the blood stream.

The risk of early onset infection is greater with prolonged rupture of the membranes, signs of chorioamnionitis (a maternal fever and tender abdomen), as well as with increasing prematurity.

In most reports the mortality from early-onset sepsis (10-30%) is higher than that from late-onset sepsis (5-10%).

Late-onset sepsis is generally much more common than early-onset sepsis. The risk of LOS is also greater with decreasing gestation and birthweight – over 20% of babies on neonatal units of 1000g birthweight or less may develop sepsis compared with less than 1% of babies with birthweight over 2000g.

Late-onset sepsis may be caused by organisms acquired from the mother but much more commonly from other babies or elsewhere in the NICU environment. We call this nosocomial or cross-infection – one baby has infection with, for instance *Klebsiella sp.*, and the organism is carried from that baby to another baby in the same NICU, usually by the staff caring for the babies.

All babies, and adults, carry organisms on their skin – we call this "normal flora". A common organism on the skin is coagulase-negative *Staphylococcus* (also called *Staphylococcus epidermidis*). In NICU babies, particularly if they are premature, this organism can cause sepsis, and in most NICUs is the commonest cause of late-onset sepsis.

What is the outcome from sepsis?

If recognised early enough most babies can recover from sepsis but they usually need to stay on the NICU for longer than they would have done otherwise.

Each sepsis episode means that scare resources are needed to treat the baby and there will be a risk of side-effects from, for instance, the intravenous line and the antibiotics.

Babies can die from *Staph. epidermidis* sepsis but the mortality is much higher with other organisms, such as Gram-negative bacteria.

Infection also contributes to long term learning and developmental problems, particularly in preterm babies, even if the baby does not have meningitis.

How to prevent infection

The incidence of late-onset sepsis can be reduced by several simple measures – **the most important of these is HAND-WASHING**
:

- Careful hand-washing on entering the unit – MUST apply to everyone

- Careful hand-washing or use of an alcohol based rinse before and after going to every baby

When the NICU is busy and crowded people sometimes say "there is not time to wash hands between every baby" but this is when the risk of cross-infection is highest. By not preventing cross-infection the NICU will get even busier with sick babies!

- Careful skin preparation /cleaning before venipuncture or placing an intravenous line

- Do not leave toys or other objects in the baby's cot – these become colonised by bacteria

- Being cared for by the mother and being fed her breast milk helps the baby become colonised by "good" organisms

- Avoid using broad spectrum antibiotics and stop antibiotics early if all cultures are negative

- Make sure that all the baby's visitors are well and **WASH THEIR HANDS**

How to prevent infection

It is important that the NICU's environmental policy is followed. Remember to check how often intravenous lines, ventilator tubing etc, might need changing according to the local policy.

Check you know what solutions you should be using to clean equipment.

- Light cleaning of incubator or cot and equipment around the baby each shift

- Use of individualised equipment, eg stethoscope or scissors. If the equipment is to be shared, then it needs to be cleaned before and after use.

Which is more common
– early-onset sepsis (that is infection in the first 48 hours of life),
or late-onset sepsis (that is infection after the first 48 hours)?

Where do the organisms causing late-onset sepsis come from?

What is the outcome from late-onset sepsis?

How can late-onset sepsis be prevented?

Are there 3 or 4 practical things you could suggest which may help reduce sepsis in your nursery?
(Please list these)

(These suggestions will go into a book for all the staff to consider)

THE END – THANK YOU
