# Supplementary material for: Capacity building of nurses providing neonatal care in Rio de Janeiro, Brazil: methods for the POINTS of care project to enhance nursing education and reduce adverse neonatal outcomes
Source: BMC Nurs. 2012 Mar 12;11:3. doi: 10.1186/1472-6955-11-3 (PMC3395837; doi:10.1186/1472-6955-11-3)
Supplement: Additional file 6 — PoC Mini-course on newborn nutrition. [file 1472-6955-11-3-S6.PDF]

MINI- COURSE

On

NEWBORN NUTRITION

Instructions:

Read each sheet and answer any questions as honestly as possible

The first sheets have four questions to allow you to give your thoughts about nutrition in babies

The next sheets give you some information about newborn nutrition

- *Why good nutrition and growth are important*
- *The advantages of breast milk and early breast milk feeds*
- *How to improve early growth in newborns*
- *How to manage milk feeds for babies in order to promote good nutrition*

The four questions are then repeated. We will not be giving marks for "right" answers but do ask you to answer all the questions to achieve a certificate showing you have completed this ***Mini-Course***

Why is good nutrition important for babies?

What is the best type of milk for babies to be fed and why?

How soon should newborns have milk (enteral) feeds started and why?

How much weight would you expect a 1000g baby (and 2000g and 3000g baby) to put on every day?

Common false beliefs

- "Babies in neonatal units don't suffer from poor nutrition"

- "Formula milk has extra nutrients, vitamins and minerals added to it, so it is better for babies than human breast-milk"

- "Starting gastric tube feeds in very preterm infants should be delayed"

Guiding Principles

Good early postnatal nutrition in preterm or sick newborns is an important aspect of care in order to promote infant growth and developmental outcomes.

Breast-milk is the ideal food for babies of all weights and gestations

Growth and Nutrition

Adequate nutrition is essential for normal growth and developmental.

We know poor *in-utero* nutrition can affect babies' outcomes, however poor or inadequate early postnatal nutrition is now also being shown to have adverse long and short term effects on both the growth and development of babies.

Evidence suggests early poor growth in babies has long lasting effects such as short stature and poor neurodevelopmental outcomes.

Good nutritional status in babies is also important as it can help improve their recovery and adaption to extra-uterine life and decrease the risk of infection and sepsis.

Poor nutrition and growth can also delay baby's discharge from hospital

The Nutritional Feeding Journey

The type, timing and route of administration of nutrition to the newborn are an important part of their nutritional management.

What are babies nutritional requirements?

When managing the type of nutrition baby receives there are 3 general things that need to be considered:

Energy (or calories)

This comes from a mixture of protein, fat, and carbohydrates. Increased calorie requirements are needed in the preterm baby, babies with respiratory disease or congenital cardiac conditions, or babies recovering from surgery or sepsis. Enough calories need to be provided to meet energy needs and achieve growth by ensuring babies have enough protein in their diet for cell building and are not breaking it down to meet energy requirements.

Minerals and solutes

These are things such as sodium, potassium, calcium, chloride, zinc, iron.
There are daily recommended intakes. Different types of enteral or parental nutrition contain different levels or can be made up to contain the required levels.

Vitamins

Vitamins are essential to normal metabolism and health. Again there are daily recommended intakes. Additional supplements may be required for certain babies

Nutrition

Calorie reserves can only sustain newborns for a short time in the absence of feeding. Nutritional management is an essential part of care to promote ongoing health and growth. Newborns in Neonatal Units can receive fluids and nutrition in 3 ways.

- **IV Fluids (dextrose):** Short term management only, IV fluids provide essential fluid & carbohydrate for hydration and blood sugar maintenance but no other form of calories to support ongoing health & growth

- **Parenteral Nutrition (PN):** This is Intravenous nutrition that provides fat, protein, carbohydrate, minerals, vitamins & calories to support growth. PN can be lifesaving and helps address the need to supply early nutrition in order to prevent under nutrition in babies unable to tolerate enteral feeds due to immaturity, disease, malformation.

- **Enteral Nutrition:** Enteral feeding (milk feeding) is considered the best and preferred way to deliver nutrition to babies.

How can we improve the early growth of babies?

Studies suggest shorter times to reach maximal nutritional intake (enteral or parental) decreases the duration of hospitalization. Enteral or milk feeds are the best way to deliver nutrition to most babies, so we need to try and safely establish milk feeds in babies as soon as possible.

Milk Feeds

- The ideal food for babies of all gestations and weights is **mother's own (unpasturised) breast-milk**

- Breast-milk is better tolerated than formula when starting enteral nutrition

- Preterm babies are shown to benefit from starting minimal enteral feeding early and they should only be stopped if there is clear signs of gastrointestinal disease

- Advancement of feeding is made on evidence of tolerating feeding

- Gastric residuals alone are not the sole reason for starting, advancing or stopping milk feeds

- The type and volume of gastric residual, plus abdominal distention, and stooling should also be considered as well as the baby's whole clinical picture!

- Careful consideration should be taken when withholding or stopping feeds, as milk feeding is very important in helping babies achieve good nutrition

<u>Minimal Enteral Feeds (MEF)</u>

Minimal enteral feeds, are also called trophic feeding, minimal enteral nutrition, or gut priming.

They may be a way to help get preterm babies onto full milk feeds sooner. Often this would be eg 0.5-1ml of fresh breast milk 4-6 hourly

Fresh breast milk contains a wide variety of growth hormones, cytokines and other factors, many of which are destroyed by pasteurisation.

Evidence suggests starting early, low volume, enteral feeds (**with fresh unpasteurised mother's own breastmilk**) may improve the development and function of the preterm infant's gut and protect against infection.

MEF have been shown to:
Improve gut motility
Improve feeding tolerance
Help in achieving full enteral feeds earlier
Improve weight gain and bone mineralisation
Decrease sepsis
Promote earlier discharge

When the baby is well the baby can be overlapped to full enteral feeds. For a <1000g the feeding would first increase to 1ml hourly and then advanced as on the next sheet.

How should we advance enteral feeds in tube-fed babies

In well preterm babies, who are tolerating feeds (and some of whom will already have been on minimal enteral feeding), the daily volume can increase as follows:

- Day 1 65 ml/kg/d
- Day 2 90ml/kg/d
- Day 3 120ml/kg/d
- Day 4 150ml/kg/d

Many small babies may need to increase to 180ml/kg/d (or even 200ml/kg/d) beyond the first week of life.

In some babies the advancement may be made over 2 days, so that 150ml/kg/d is reached on day 7.

In general, for babies <1000g, start with hourly feeds and increase feeds by eg 1ml/hr each 4 hours.

For babies 1200-1400g, the interval can be 2 hourly. Increase feeds by eg 2ml/hr each 4 hours.

For babies >1400g, the interval can be 3 hourly. Increase feeds by eg 3 ml/hr each 6 hours.

- **<u>Types of milk available for enteral feeding</u>**

- **Mother's own unpasturised expressed breast milk**
  (this is the gold standard and is the recommended milk for enteral feeding due to the unique properties of breast-milk)

- **Banked breast milk**
  (this can be used if there is not enough of mothers own milk as an alternative to formula. It allows babies to benefit from some of the unique properties of breast-milk that can't be found in infant formula. Some of the properties of breast milk are affected during pasturisation)

- **Fortified breast milk**
  (designed and recommended for use with preterm infants. Whey protein concentrate powder that can be added to breast milk to provide additional calories, protein, vitamins, minerals)

- **Standard infant formulas**
  (often just called milk mixture)

- **Special preterm formulas**
  (have a higher protein, mineral content than standard formula designed for use in preterm infants to provide additional nutrition for growth needs, has a higher kcal content than standard formula)

Breast Milk

Human milk is a special and unique food for babies that not only meets nutritional needs of babies, but also to give immune protection and promotes development. Breast milk has many immunological, nutritional and gastrointestinal advantages and supports growth, development and survival by:

- **Protection for babies from infection/sepsis**
  (human milk contains immunological factors that help protect newborn babies)
- **Promotes gastrointestinal health & function and offers some protection from necrotizing enterocolitis**
  (there are several factors present in human milk that are important to gut growth, maturation and immunity. Formula feed babies have been shown to develop NEC more often than babies receiving only human milk)
- **Excellent nutritional value with increased feeding tolerance and absorption of nutrients**
  (evidence suggest nutrients from human milk are better absorbed, it is better tolerated and babies have been shown to establish enteral feeding sooner than with formula milk)
- **Neurodevelopmental advantages**
  (there is evidence to suggest human milk may offer some neurodevelopmental advantages for babies, both with IQ & developmental outcomes)
- **Provides growth regulatory effects**
  (human milk contains growth factors and hormones)
- **Decreased risk of later allergy**
  (lower incidence allergy shown in human milk fed babies at 18 months)

<u>How can we tell if babies are tolerating feeds?</u>

Assessment of signs of feeding tolerance is an important part of milk feeding management. It helps in deciding if feeds can grade up in volume or frequency, or if feeding complications are occurring. Assessments that should be made are:

- **Gastric Residuals:** Aspirate feeding tubes 4 - 6hrly. Assess residuals for volume, colour, digestion
- **Vomiting:** Is there any? If so when, how much, how often
- **Abdominal distention:** Present? Absent? Tense? Tender?
- **Stools:** How often, type, any blood in the stools?
- **Apnoea/bradycardia:** Are there any? When do they occur? Are they more frequent?
- **Infant behaviour/appearance:** How does the baby look? pink, active?, pale mottled?

Possible indications for stopping feeds

- Abdominal distension/tenderness/rigidity
- Lethargy, hypotonia, apnoea
- Blood in stool, or aspirates
- Bilious vomiting
- Large gastric residuals

How can we help babies tolerate milk feeding and promote nutrition?

- Non nutritive sucking with feeds for babies that are too ill or immature to attempt oral feeding yet
- Prone or side-lying during or after feeding helps with feed tolerance
- Reduction of stress and stimulation before or during feeding
- Adequate pain control
- Attention to temperature control and energy expenditure
- Supporting mothers and helping them with expressing breast milk
- Kangaroo mother care
- Once babies are ready for oral feeding breastfeeding is the ideal method of feeding and nurturing infants and promoting nutrition

Kangaroo Mother Care

Skin to skin contact (even for a brief period) between mother and baby is an essential part of care for babies in neonatal units. It has many benefits for both but is very important in the nutritional management of babies as it:

- Helps mothers with milk production
- Assist with later establishment and duration of breastfeeding
- Has a stabilizing effect on babies cardio-respiratory parameters & behaviour (less crying, better sleep)
- Enhances growth in babies

How can we monitor the growth of babies?

Babies growth is a practical and valuable way to help determine if nutritional goals are being met. Growth should be assessed on weight, head circumference, and length.

Ideally, these measurements should be plotted on appropriate growth charts.

- **Weight** (every 2 - 4 days)
  initial weight loss of < 10% of body wt desirable
  and regaining birth-weight within 2-3wks
  Aim for : Wt gain of 10g per day for a 1000g baby; 20g per day for a 2000g baby; 30g per day for a 3000g baby.
  (These are *in utero* growth rates, ie 10-15g/Kg/d)

- **Head circumference** (measure and plot weekly)
  Use paper or non stretch plastic tape for accuracy
  Aim for : > 0.9cm growth per week

- **Length** (Measure and plot weekly)
  Reflects skeletal and organ growth
  Aim for : > 0.9cm growth perweek

If babies show signs of poor growth what can we do?

Consider:
The type of feed being given
Increasing feed volumes
Increasing calories in feeds
Other factors that may increase calorie expenditure such as thermal instability, overstimulation, sepsis, feed intolerance

Why is good nutrition important for babies?

What is the best type of milk for babies to be fed and why?

How soon should newborns have milk (enteral) feeds started and why?

How much weight would you expect a 1000g baby (and 2000g and 3000g baby) to put on every day?

Are there 3 or 4 practical things you could suggest which may help improve babies' nutrition in your nursery?
(Please list these)

(These suggestions will go into a book for all the staff to consider)

THE END – THANK YOU
