# Supplementary material for: Capacity building of nurses providing neonatal care in Rio de Janeiro, Brazil: methods for the POINTS of care project to enhance nursing education and reduce adverse neonatal outcomes
Source: BMC Nurs. 2012 Mar 12;11:3. doi: 10.1186/1472-6955-11-3 (PMC3395837; doi:10.1186/1472-6955-11-3)
Supplement: Additional file 7 — PoC Mini-course on temperature control in the newborn. [file 1472-6955-11-3-S7.PDF]

MINI - COURSE
On
TEMPERATURE CONTROL IN THE NEWBORN

<u>Instructions:</u>

Read each sheet and answer any questions as honestly as possible

The first sheets have four questions to allow you to give your thoughts about temperature control in babies

The next sheets give you some information about temperature control the newborn
- *How do newborns try to keep their body at the right temperature*
- *Why do newborns have more difficulty than adults maintaining the right body temperature*
- *How does the environment affect temperature?*
- *Why is being too cold or too hot harmful for newborns?*
- *How can we help newborns keep the right temperature*

The four questions are then repeated. We will not be giving marks for "right" answers but do ask you to answer all the questions to achieve a certificate showing you have completed this ***Mini-Course***

How do babies try to raise their temperature when they get cold?

Why do babies have more difficulty maintaining their temperature than adults?

What signs and symptoms does a baby show if it is too cold?

The environment affects a baby’s temperature. Can you name one thing in the environment that would cause a baby to get cold and what you would do to prevent it?

Common false belief

"Babies can maintain their temperature just as well as adults"

Guiding Principles

Neonates, term and preterm, experience significantly more challenges maintaining their temperature than adults and are susceptible to heat loss.

Hypothermia in neonates is associated with increased morbidity and mortality.

Thermal management of babies is a vital and critical part of Neonatal Care.

Care is aimed at maintaining the neutral thermal environment (the environmental temperature at which minimal rates of oxygen consumption or energy expenditure occur)

Heat Balance and Temperature Control

Maintaining a core body temperature within a normal narrow range is essential to survival

Temperature is maintained by balancing the heat produced and heat lost despite changes in the environment.

Body temperature is regulated by changes in metabolism, motor tone and activity, vasomotor and sweating activity, to produce either heat gain or heat loss.

How do babies try loose or gain heat to keep the right body temperature?

Babies try to loose or gain heat in the following ways.

Babies try to loose heat by:

Decreasing their activity to try and decrease heat production

Vasodilation (limited response)

Sweating (limited response)

Babies try to produce heat by:

Increasing their metabolic rate

Non shivering thermogenisis
(babies have special stores of brown fat which they can metabolise to produce heat, these stores are limited)

Increasing their motor tone & activity (limited response)

Vasoconstriction (limited response)

Why do babies have more trouble than adults maintaining temperature control?

Babies have a lot more difficulty than adults maintaining the right temperature. They tend to loose body heat quickly and easily and have more difficulty generating heat to warm up. Listed below are some of the reasons why.

- Less insulation - babies have a thinner layer of subcutaneous fat than adults so loose heat more readily

- A large surface area to body mass ratio - babies have more area to loose heat from than adults

- Greater evaporative heat loss - because of immaturity of their skin compared to that of adults, babies can loose a lot of heat and water through their skin

- Less ability to produce heat through shivering, activity, & changes in position - babies rely on non shivering thermogenisis or brown fat metabolism for heat production.

- Less ability to vasoconstrict or dilate to gain or loose heat

- Limited stores of metabolic substrates to use for heat production

- Less control of the regulation of their temperature related to more immature central nervous system.

Why can being cold be dangerous to a baby?

Babies are very susceptible to heat loss. The effects of cold on babies can be very serious and being cold is known to increase morbidity and mortality.

Most of the effects seen when babies get cold are the result of two major changes that happen in response to cold stress.

These are:
An increase in a babies metabolic rate &
An increase in their oxygen consumption.

Both of these things can also make babies more *acidotic*, which also effects their body functioning.

HYPOGLYCAEMIA AND RESPIRATORY DISTRESS are two major problems that can happen to babies if they get too cold.

How can being cold cause hypoglycaemia?

Hypoglycaemia is a major problem that can occur when babies are too cold and is a result of the baby increasing their metabolism to try and correct their body temperature.

In an attempt to warm up by increasing their metabolism, babies use glucose. This can lead to them using up their limited stores of glucose and can then result in them becoming hypoglycaemic.

Hypoglycaemia may be associated with **seizures and brain damage.**

Also if babies continue to be in an ongoing situation where they are too cold, they use calories for metabolism versus growth. This can lead to large postnatal weight losses or **poor weight gain.**

How can being cold cause respiratory distress, an increase in oxygen need and hypoxia?

Respiratory distress, an increase in oxygen need and hypoxia can occur when babies are too cold and is the result increased oxygen consumption by baby's body.

When babies become cold their need for oxygen increases, as it is the fuel the body needs to drive actions the baby takes to try and warm themselves up and correct their body temperature.

To supply the extra oxygen needed babies increase their respiratory rate and effort. Sometimes more oxygen is needed than babies can get and this can result in respiratory distress and/or an increase in or need for oxygen and/or hypoxia.

Surfactant production is also affected when babies are cold and this can contribute to their respiratory distress as well

SERIOUS THINGS CAN HAPPEN BECAUSE A BABY HAS GOT TOO COLD!

WE KNOW BABIES CAN HAVE TROUBLE KEEPING THE RIGHT TEMPERATURE. THIS IS WHY IT IS SO IMPORTANT TO HELP BABIES KEEP THE RIGHT BODY TEMPERATURE

What can happen if babies get too hot?

Although we worry about the effect of cold on babies, there are also dangers from overheating and being to hot can also increase morbidity and mortality

If babies get too hot they can have:

Increased fluid losses from evaporation and sweating.

Increased weight losses

Being hot also increases their metabolism and respiratory rate as they try to cool down

Babies can also have apnoeas from being too hot

How does the environment affect a baby’s temperature?

The environment can have a big impact on a baby’s temperature and babies are dependent on their caregivers to help provide them with the right environment to help them keep the right temperature.

Babies can loose or gain heat in the environment in four major ways and it is important as caregivers to know what they are so we can help provide babies with the environment that prevents them from either getting too hot or too cold.

The ways babies can loose or gain heat by are:

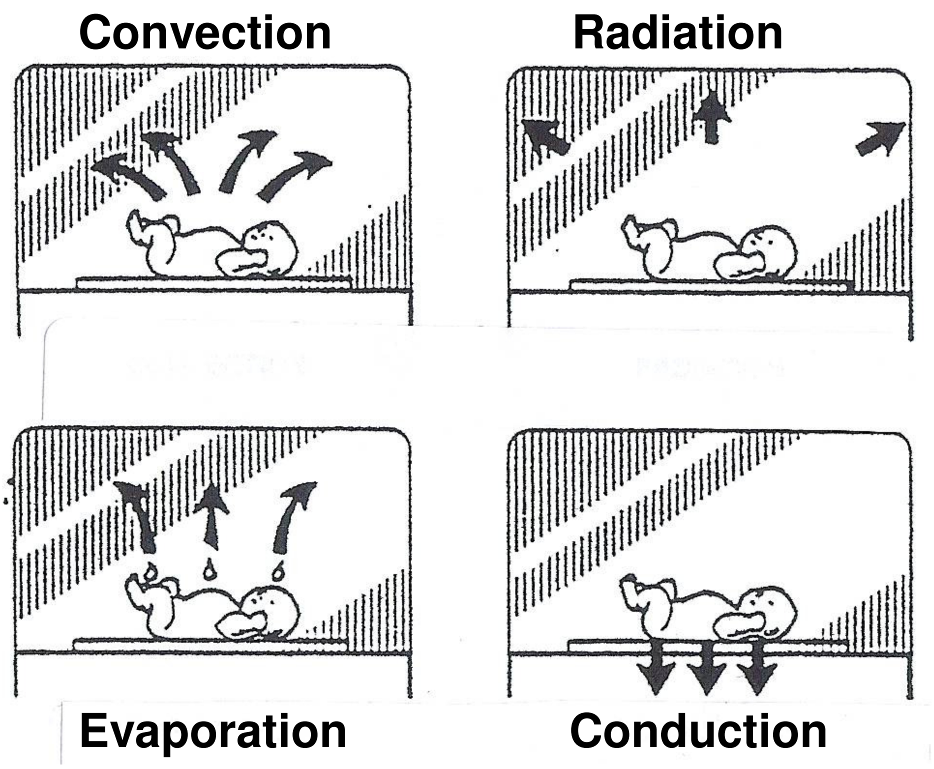

- **Convection is:** the transfer of heat to the air moving around and across the body

- ***Radiation i*s:** the transfer of heat between two surfaces that are not in direct contact

- **Evaporation is:** heat loss through energy used in the conversion of a liquid into vapour

- **Conduction:** is the transfer of heat between two solid objects that are in direct contact

Convection

Some examples of when babies can loose heat by convection include:

- drafts from air vents, windows, doors, fans
- cold oxygen flow

Can you think of others?

- cool rooms, corridors,
- cool incubator

Radiation

Some examples of when babies can loose (or gain) heat by radiation include:

- from cold incubator walls
- being placed near cold walls or windows

Can you think of others?

- from phototherapy lights

Evaporation

Some examples of when babies can loose heat by evaporation include:

- at birth when amniotic fluid evaporates
- wet body/hair after bathing

Can you think of others?

- the application of solutions, lotions to infant
- water loss from lungs
- increased insensible water loss through skin in VLBW infants, infants with skin damage, abdominal wall defects such as gastroschisis

Conduction

Some examples of when babies can loose heat by conduction include:

- being placed on cold linen or towels
- being left on wet linen or towels

Can you think of others?

- being placed on cold scales
- being placed on cold xray plates
- being placed in cold incubators or on cold heat shields

How can you tell if a baby may be too cold or too hot?

Babies can present with some of the signs and symptoms listed below if they become to cold or to hot.

It is always important to check a baby's temperature if they are showing any of these

- Apnoea, tachypnoea, respiratory distress
- Cyanosis, oxygen requirement
- Bradycardia, poor perfusion
- Hypoglycaemia
- Restlessness, irritability, jitteriness
- Lethargy
- Feeding Intolerance
- Acidosis

REMEMBER IT IS NOT JUST THE ENVIRONMENT THAT CAN CAUSE A BABY TO GET TOO HOT OR TOO COLD.

IF A BABY HAS AN UNSTABLE TEMPERATURE OR IS TOO HOT OR COLD, DO NOT FORGET ABOUT **INFECTION** AS A POSSIBLE REASON

How can we prevent babies from getting too cold or hot ?

Monitoring babies temperatures regularly and if the are showing any signs or symptoms of thermal stress.
Aim for an axilla temperature of 36.5 - 37.0 C

Actively manage the environment for babies to prevent heat loss or overheating.

Remember the mechanisms of *convection, conduction, evaporation, radiation,* that babies loose or gain heat by and identify the sources that can cause these for babies

Listed below are some simple ways to prevent babies from getting cold. Can you think of any others?

- Dry the baby immediately after birth or bathing
- Use pre-warmed towels to dry baby
- Pre-warm incubators/heat shields before use
- Use incubators and heat shields to manage the temperature for sick and immature babies
- Pre-warm linen & clothing
- Warm solutions used on baby's skin
- Avoid putting babies onto any cold surfaces
- Avoid putting babies near drafts
- Nurse babies in warm rooms
- Kangaroo care
- Promptly remove change any wet linen or clothing
- Regularly monitor baby's temperature adjust environment to maintain correct temperature

Labour Ward Management

It is very common for babies to get cold in Labour Ward.

This is particularly a major problem for very low birthweight and premature babies when the resulting acidosis makes hyaline membrane disease more severe.

It is important to keep the Labour Ward warm.
Preterm babies should be dried and covered as soon as possible after birth.

For babies of <28 weeks gestation or <1000g it has been shown that placing the baby under an occlusive plastic wrap, without first drying, can very effectively prevent hypothermia.

Many units find it easiest to put the baby directly into a plastic bag ("freezer" bags work well) straight after birth. The baby can be observed through the bag and the heart rate listened to.

How do babies try to gain heat when trying to keep their body at the right temperature?

Why do babies have more difficulty maintaining their temperature than adults?

What signs and symptoms does a baby show if it is too cold?

The environment affects a baby’s temperature. Can you name one thing in the environment that would cause a baby to get cold and what you would do to prevent it?

Are there 3 or 4 practical things you could suggest which may help manage a baby's temperature control in your nursery?

(Please list these)

(These suggestions will go into a book for all the staff to consider)

THE END – THANK YOU
