# Supplementary material for: Capacity building of nurses providing neonatal care in Rio de Janeiro, Brazil: methods for the POINTS of care project to enhance nursing education and reduce adverse neonatal outcomes
Source: BMC Nurs. 2012 Mar 12;11:3. doi: 10.1186/1472-6955-11-3 (PMC3395837; doi:10.1186/1472-6955-11-3)
Supplement: Additional file 8 — Mini-course on supportive care in the newborn. [file 1472-6955-11-3-S8.PDF]

MINI - COURSE
On
SUPPORTIVE CARE
IN THE NEWBORN

Instructions:

Read each sheet and answer any questions as honestly as possible

The first sheets have four questions to allow you to give your thoughts about supportive care in babies

The next sheets give you some information about Supportive Care of the newborn

- *How the preterm and unwell baby can be affected by NICU environment*
- *How babies communicate through behaviour to indicate if the environment is stressful for them*
- *How handling the newborn can affect them*
- *How we can modify our care-giving environment and practices to support the baby in the neonatal unit*
- *The importance of involving the family in the caring for their baby in the Neonatal Unit*

The four questions are then repeated. We will not be giving marks for "right" answers but do ask you to answer all the questions to achieve a certificate showing you have completed this ***Mini-Course***

List two aspects of the Neonatal environment that can be stressful for a baby.

List two behaviours that a baby may display and which indicate that it is stressed by the neonatal environment.

There are many things we can do to help reduce stress and support a baby's development when they are being cared for in the Neonatal unit. Name one thing you can do.

Kangaroo Care has many benefits for both mother and baby: name one benefit for the baby and one for the mother.

Common false belief

"The only thing which matters with a preterm baby is to deal with the medical problems – there will be plenty of time later for the baby to develop normally"

Guiding Principles

With sick preterm infants –
ONE THING LEADS TO ANOTHER

Babies can quickly become unstable
so all aspects of care are equally important

Supportive Care or Developmental Care is the use of a range of practices, which are now being incorporated into neonatal intensive care in order to reduce any adverse impact of the neonatal unit on the developing baby and their family and to support and promote optimal neurological and behavioural development and outcomes.

Key aspects of Supportive Care are:

Family Centered Care:
Strategies and interventions to facilitate bonding and foster parent-infant interaction.

Environmental Manipulation & Modification:
Interventions to reduce the stress/impact of the physical NICU environment on the preterm and sick infant

Handling and Positioning Practices:
Strategies and interventions to minimize the stress of handling and care-giving practices. Positioning that promotes neuromuscular development.

Behavioural Organization Assessment & Promotion:
Recognizing and responding to a baby's behavioural cues. Organizing individualized care to help the baby maintain physiological and behavioural "stability" and minimize stress and pain.

1. Family Centered Care

Why is it important?
"Isn't it a lot of extra work and isn't my job just to medically look after the baby?"

When caring for babies in the NICU it is essential to involve the family. Babies belong to the family unit not the neonatal unit. What you do to one affects the other and vice versa. So when we care for a baby in the NICU we also need to care for and involve the family.

Infant growth and development is tied to parent-infant attachment and interaction. The parent-infant relationship is biologically and psychologically essential for survival and development of the infant.

Multiple adverse outcomes have been associated with preterm birth/NICU experience, such as

- heightened parental stress
- negative parent infant interaction
- adverse child developmental and behavioural outcomes

If we are to promote infant outcomes and support infant development we have to support families and facilitate the process of attachment and bonding and parent-infant interaction.

What are the core elements of Family Centered Care and how can I practice it in everyday care?

Family Centered care is relationship based care and involves

- **P**arental Involvement
- **P**arental Participation
- **P**artnership with Parents (families)
- **S**hared Care (collaboration)

Healthcare professionals have a vast range of skills and knowledge that are needed to help a NICU baby not only to survive, but survive with the best possible outcomes.

However, babies also need their parents and they need their parents to get to know them and be involved with their care. We need to share our work-space, time, knowledge and information with the baby's parents.

This starts with simple things such as:

- Being welcoming & respectful of their role as a parent
- Giving information freely and truthfully
- Involving them in decision making about the healthcare of their baby
- Actively teaching parents about their baby and how to gently handle them and be involved with physical care-giving such as temperature taking, nappy changing, bathing and weighing.

2. Environmental Manipulation and Modification

How can the Neonatal Unit environment impact on the baby?

It is well recognized that the Neonatal Unit environment can have a big impact on babies and has the potential to affect short and long-term outcomes and development.

The sick or preterm baby is in a situation of mismatch with the Neonatal environment and often doesn't have the developmental capability to deal with the stresses of the neonatal unit.

Management of the neonatal environment is a core element in "supportive care". Environmental management involves a broad range of interventions and strategies designed to modify the physical and care giving environment of the NICU and to decrease the stressful impact on babies.

Supportive Care also involves and is dependent on caregivers being able to recognize and respond to the behavioural "CUES" or communications babies send out to tell caregivers (staff and parents) how they are coping with what is going on around them or to them!!

What aspects of the NICU environment affect the baby?

All aspects of the physical environment have the potential to impact on a baby and to be an unpleasant experience.

Listed below are some of the aspects of the environment that can be potential sources of stress and cause a negative response in a baby. These are things we try to manipulate or modify so they are less stressful.

Noise
Light
Smell
Taste
Touch
Handling
Positioning
Pain Management

Caregivers and care-giving practices have a significant impact on babies so careful attention needs to be paid to this aspect of care as well as the physical environment.

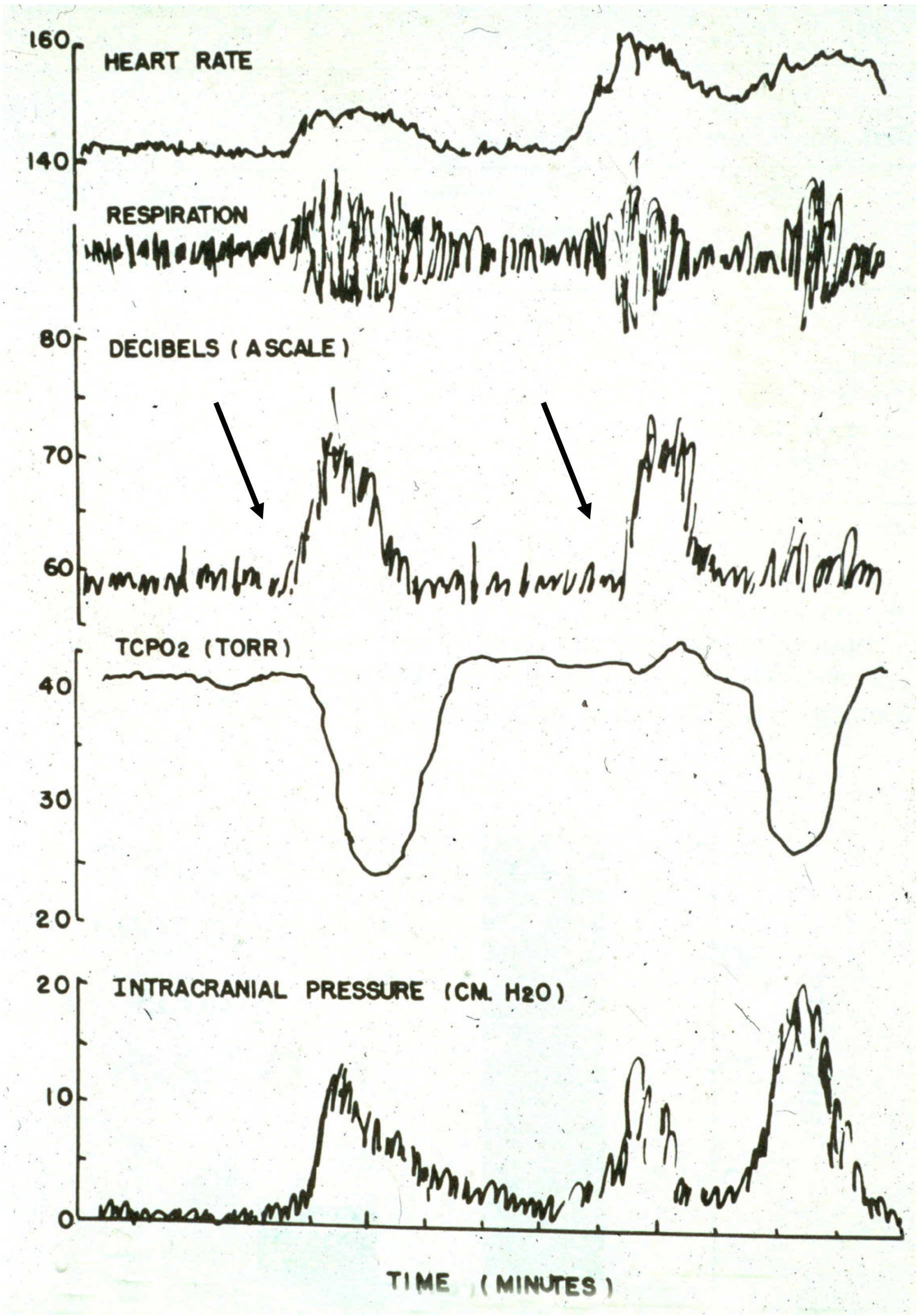

Adapted from Long JG, et al. Pediatrics 1980; 65: 143-5

Look at this picture. The baby has a stable heart rate and respirations until the loud noise (↘) when the heart rate rose, respirations increased, oxygen tension fell and intracranial pressure rose.
Babies become unstable very easily!

How can we change the environment so it is less stressful for babies?

By identify potential sources of environmental stress for babies and modifying the physical and the care giving environment you can help reduce this stress. Every NICU is different and the first step is to identify what things in your NICU and practice are potential sources of stress. Below are some examples of questions to ask yourself to help you identify potential environmental stressors

Noise: What causes noise in your NICU and when? (People and equipment!)

Light: What are the sources of light and can you adjust the lighting levels?

Taste: What tastes do babies experience? Do they get any oral medicines or mouth cares with any solutions; do they get drops of EBM in their mouths when awake and receiving NGT feeds?

Smell: What smells are baby's exposed to? Staff perfumes, cleaning solutions?

Touch/handling: Who touches/handles the babies? How often? What for?

Postioning: What positions are babies nursed in? Do you use nests or boundaries to help maintain them in position and keep them calm?

Pain: What procedures and practices do you do that could be painful? What medications do you use for pain relief and when?

How can we tell if the environment is affecting the baby?

Babies communicate primarily through BEHAVIOUR.
Behaviours tell us that babies are finding the environment stressful or they are coping with the environmental stimuli around them – so they send signs of **STRESS or STABILITY**
Understanding and responding to infant behaviour is key to supporting infants and their development
The preterm baby is dependent on his caregivers to read the behavioural signs or **CUES** the baby is sending

You can observe behavioural cues in the systems listed below and use them to quide you in caring for and planning the care of individual babies to try and keep them as “stable” as possible.

- **Autonomic (physiological):** The HR, respirations, colour, visceral responses
- **Motor:** The tone, posture, activity, movement,
- **State Organisation:** The state of consciousness, i.e. the range of sleep-wake states.
- **Attention/Interaction:** The baby’s availability, alertness, robustness of interaction. Babies can usually only manage this when the three systems above are stable
- **Self-Regulatory:** The baby’s ability to return to or maintain stability/organization in their autonomic/motor/state system when exposed to environmental stimulation. When babies are sick or immature they often need careful input and management from caregivers to help them achieve this.

What are some of the signs of stress or stability?

Note: Changes in a baby's functioning may occur during or following stimulation either from the environment or handling

System	Signs of Stress	Signs of Stability
**Autonomic:**	Unstable heart rate/BP<br><br>Changes rate or character of breathing e.g. apnoea, tachypnoea<br>or decrease in oxygen saturations<br><br>Change in skin colour (pale, mottled, dusky)<br><br>Visceral responses like gagging, hiccupping, spilling, increased gastric residuals	Stable heart rate/BP<br><br>Regular respiratory pattern<br>Stable oxygen saturations<br><br>Pink stable skin colour<br><br>Stable digestion
**Motor:**	Frantic disorganised movements<br><br>Extension or limpness of arms or legs<br><br>Finger toe splaying<br><br>Facial frowning, grimacing	Lying quietly or smooth movements<br><br>Flexed or tucked position
**State Organisation**	Poorly defined sleep, wake states<br>Low level alertness, dull, glassy eye look<br>Hyper alert, e.g. wide eyed with panicked worried look<br>Active gaze averting e.g. closing eyes or looking away	Well defined sleep wake states<br><br>Focused alertness, including<br>open face doesn't look worried

3. Handling and Positioning Practices.

How can handling and care giving practices effect babies?

Adaptation and infant development of the preterm or sick infant are influenced by care and handling practices

How should we position babies?

Positioning the preterm infant is a very important aspect of care. Good positioning practices promote neuromotor development and can have a positive affect on both short and long term outcomes for babies. Correct positioning practices can:

- Prevent development of skeletal & postural deformities that have a later impact on development
- Promote the development of physiological flexion
- Positively affect physiological and behavioural parameters e.g. better sleep, more stable oxygenation.
- Reduce stress, encourage relaxation and digestion
- Prone positioning can increase oxygenation when compared to supine
- Encourage development of flexor tone, hand to mouth activity, midline orientation, all of which are important to later development
- Promote growth by increase feelings of security self control

Good positioning involves:

- Monitoring a baby's physiological and behavioural response to their position
- The use of nesting or boundaries to help maintain babies in position.
- Utilizing the variety of positioning options, lateral, prone supine (supine should be avoided as a the predominant position for preterm babies)
- Ensuring that, whatever position the baby is in, they are symmetrically positioned in flexion with hands near head next to face mouth.

Kangaroo Care

Kangaroo Care is an important aspect of neonatal care. Babies are handled many times and for many reasons. Kangaroo care offers the baby the opportunity to experience some “pleasurable” TOUCH experiences.

Research shows many benefits for both baby and mother. And kangaroo care does not have to be confined to mother and baby only, fathers should also be encouraged with it.

Any gestation of infant being cared for in a neonatal unit can benefit from kangaroo care. Attention needs to paid to the environment around the baby when the baby is having skin to skin contact to help both the baby and the mother/father get the most benefit from it, e.g. quiet, private, and warm.

Benefits of Kangaroo Care include:

Promotes/enhances breastfeeding:
Initiation / exclusivity / duration / milk production

Psychosocial:
Improved parental feelings and decreased anxiety.
Improved attachment, more positive interactions

Behavioural:
Improved sleep integrity / decreased crying

Physiological:
Stabilizing effect on cardiorespiratory parameters

4. Behavioural Organization Assessment and Promotion

This brings together all the themes above:

- Family centred care
- Environmental manipulation and modification
- Handling and positioning practices

But recognizes that babies are all different so there will need to be an individualized care plan for each baby and their family.

List two aspects of the Neonatal environment that can be stressful for a baby.

List two behaviours a baby may display that indicate it is stressed by the neonatal environment.

There are many things we can do to help reduce stress and support a baby's development when they are being cared for in the Neonatal unit. Name one thing you can do.

Kangaroo Care has many benefits for both mother and baby, name one benefit for the baby and one for the mother.

Are there 3 or 4 practical things you could suggest which may help manage a baby's temperature control in your nursery?

(Please list these)

(These suggestions will go into a book for all the staff to consider)

THE END – THANK YOU
